# Lyophilization of ASFV vaccine candidate ASFV-G-ΔI177L offers long term stability

**DOI:** 10.1038/s41598-024-80170-2

**Published:** 2024-12-30

**Authors:** Nallely Espinoza, Edward Spinard, Ayushi Rai, Elizabeth Ramirez-Medina, Alyssa Valladares, Amanda Meyers, Manuel V. Borca, Douglas P. Gladue

**Affiliations:** 1 Plum Island Animal Disease Center, Agricultural Research Service, USDA, Greenport, NY 11944 USA; 2 National Bio and Agro-Defense Facility, Agricultural Research Service, USDA, Manhattan, KS 66506 USA; 3 Oak Ridge Institute for Science and Education (ORISE), Oak Ridge, TN 37830 USA; 4 Kansas State University, Manhattan, KS 66506 USA; 5 Seek Labs, Utah Salt Lake City, 84103 United States

**Keywords:** ASFV, Vaccine, Lyophilization, African swine fever, Vaccines, Virology

## Abstract

For over a century African swine fever (ASF) has been causing outbreaks leading to devastating losses for the swine industry. The current pandemic of ASF has shown no signs of stopping and continues to spread causing outbreaks in additional countries. Currently control relies mostly on culling infected farms, and strict biosecurity procedures. Recently a vaccine, ASFV-G-ΔI177L was approved for use in Vietnam. In this study we evaluate the long-term stability of lyophilized ASFV-G-ΔI177L. Understanding the stability of different formulations of vaccines is information necessary for deployment of vaccines to ASF outbreak areas, particularly those that do not have a reliable well established cold chain to ensure conservation of vaccine quality. In this report, we determined that ASFV-G-ΔI177L, when lyophilized under specific conditions, is stable for up to one year at 4 °C, with similar vaccine titers after storage. Next-generation sequencing analysis also determined that lyophilization and long-term storage under these conditions had no effect on the genome of ASFV as the genome remained genetically identical to the original non-lyophilized form.

## Introduction

First reported in 1921 in Kenya^1^, African swine fever (ASF) is a deadly contagious hemorrhagic disease of domestic and wild pigs, and can be transmitted either with animal contact or through a soft tick intermediate carrier. The causative agent, ASF virus (ASFV), is a large DNA virus belonging to the family Asfaviridae with a large dsDNA genome ranging from 170 to 192 kb encoding 150 to 200 proteins^2^. Historically, ASF has caused, on a regular basis, outbreak within Africa, and in some occasions sporadic outbreaks outside of Africa that were resolved locally without continuing to spread worldwide. The current ASF pandemic is a result of the 2007 outbreak of ASF occurring in the Republic of Georgia^3^ that has continued to spread every year to additional countries, remaining initially restrained to the Eastern hemisphere until 2021 when an ASF outbreak occurred in the Dominican republic^4^.

ASFV vaccine candidate ASFV-G-ΔI177L a live attenuated Genotype 2 vaccine based on the deletion of the I177L gene in ASFV^5^. This vaccine been shown to be a safe and efficacious vaccine against different isolates of the pandemic strain and in different breeds of pigs^6^. ASFV-G-ΔI177L efficiently produce protection when administered either intramuscularly or oral-nasally^7^. Safety studies, including reversion to virulence studies^8^, have been completed successfully resulting in approval for use in Vietnam, making the ASFV-G-ΔI177L vaccine one of the two first commercially produced vaccines for ASF. For a review on ASF live attenuated vaccine candidates, see Gladue, et al. 2022^9^.

Even considering the availability of an effective vaccine, ASF will continue to cause outbreaks in many geographical areas where there is not an established a reliable cold chain, causing stability problems with frozen vaccines. The activity of a live attenuated vaccine depends on their effectiveness as well as on the virus stability during storage and transportation, with lyophilization being a widely accepted method to increase stability particularly for live attenuated vaccine strains. In this report, we evaluate for the first time, the stability of a ASFV live attenuated vaccine after lyophilization using different conditions. Here we report that the ASF vaccine ASFV-G-ΔI177L, under various lyophilization conditions, can remain stable for up to one year without the need for ultra cold storage.

## Results and discussion

### Determining optimal Lyophilization parameters for the ASFV-G-ΔI177L vaccine

To determine the optimal parameters to protect the live attenuated vaccine from potential deleterious factors during the lyophilization process we tested different lyophilization conditions for the ASFV-G-ΔI177L vaccine using different drying conditions (Table 1). At the end of the drying process the temperature was set at 4 °C until the samples could be capped and removed.

**Table 1:** Different conditions tested during the process of lyophilization of ASFV-G-ΔI177L.

		Cycles (s)		Vacuum		Ramp Rate		Shelf Temperature		Hold Time hrs.		
Condition 1		Drying		0.40 mBar		0.1		-50° C		2		
Condition 2		Drying, Cooling		0.40 mBar		0.1		-50° C		2		
				0.40 mBar		0.1		0° C		0.5		
Condition 3		Drying		0.40 mBar		0.1		-50° C		2		
Condition 4		Drying, Cooling		0.40 mBar		0.1		-50° C		4		
				0.40 mBar		0.1		0° C		0.5		

The survival rate of ASFV-G-ΔI177L after lyophilization was tested for each condition. Samples were prepared by a dilution 1:5 of ASFV-G-ΔI177L (500uL I177L and 20mL media) with a final concentration of 4.66 HAD_0_/ml. The dilution was inoculated on 20 dark amber vials which were placed immediately at -70 to avoid any titer loss. The ASFV strain Georgia 2010 (ASFV-G), diluted for a final concentration of 5.0 HAD_50_/ml, was prepared following a similar process. For the lyophilization, each condition was programmed and used until the lyophilizer shelf temperature was reached. Three vials were used for each condition tested and a set of DMEM culture medium was used as control for each run. Once the program had finished, vials were rubber-stoppered under a vacuum and capped.

**Fig. 1:**
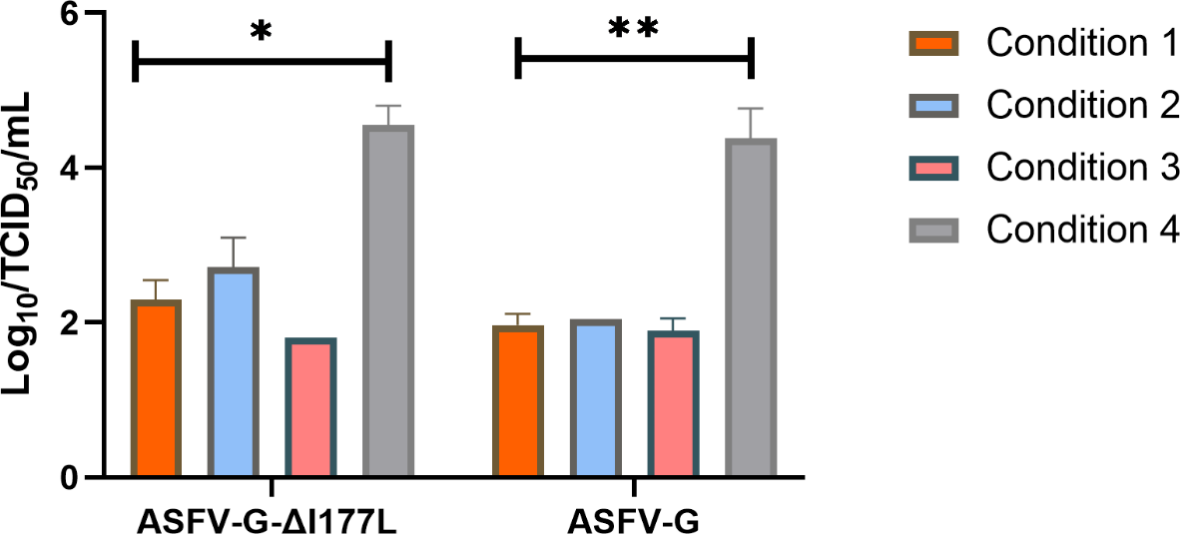
Evaluating different lyophilization conditions for ASFV-G-ΔI177L.

After storage at -70^o^C the survival of ASFV-G-ΔI177L was titrated in primary swine macrophages (Fig. 1). In comparison to the stock of virus, lyophilization conditions 1–3 produced a decrease in viral titers of more than 2 logs., Conversely, the trial 4 produced only an average decreased in virus titers of 0.31 log loss of vaccine virus mediating a better stabilization of the ASFV-G-ΔI177L vaccine. The additional cooling step in Trial 4 increased stability of the lyophilized vaccine and was selected as drying conditions for the ASFV-G-ΔI177L vaccine. As ASFV-G-ΔI177L was stable for lyophilizartion condition 4 we used condition 4 for further analysis.

### Evaluation of lyophilization stabilizers for ASFV-G-ΔI177L for short term storage

Cryoprotectants are complex reagents that prevent denaturation in vaccines during the freeze-drying process^9^. The use of cryoprotectants can potentially protect viral-coated proteins. Any protein denaturation or damage to the structural integrity could impact the activity and efficacy of any live attenuated vaccine. To protect the stability of ASFV-G-∆I177L, we proceeded to test different cryoprotectants. This step was done in two phases, the first one was to test different formulations to find the one that would protect ASFV during the freezing drying process and the second one was to test among the most protectant formulation storage at a different ratio under different storage temperatures.

In the first part, we tested six different formulations as a way of screening (Table 2).

**Table 2:** Stabilizer formulations.

Stabilizer formulation		
F1	Sorbitol	10%
	sucrose	7%
	BSA	4%
F2	sorbitol	10%
	sucrose	7%
	glycine	4%
F3	Sorbitol	10%
	sucrose	7%
	FBS	9%
F4	Sorbitol	10%
	sucrose	7%
	trehalose	4%
F5	trehalose	4%
	FBS	9%
	Sorbitol	10%
F6	skim milk	10%
	DMEM	

To further improve the stability and protective effect of the vaccine, we used formulations based on trehalose, sucrose, and sorbitol. Saccharides like sucrose and trehalose had been proven to be good stabilizers during freeze-drying (3). It had been hypothesized that sucrose and trehalose protect live viruses by replacing the hydrogen bonds with glass, thus preventing damage to the membrane and membrane proteins caused by ice crystals (2). On the other hand, Sorbitol in combination with gelatin has been demonstrated to be a good stabilizer for different viruses (4,5). Furthermore, we used BSA, FBS, and Skin Milk as protein stabilizers. The use of animal-derived compounds has been used for the process of lyophilization of attenuated vaccines to prevent inactivation (6).

**Fig. 2:**
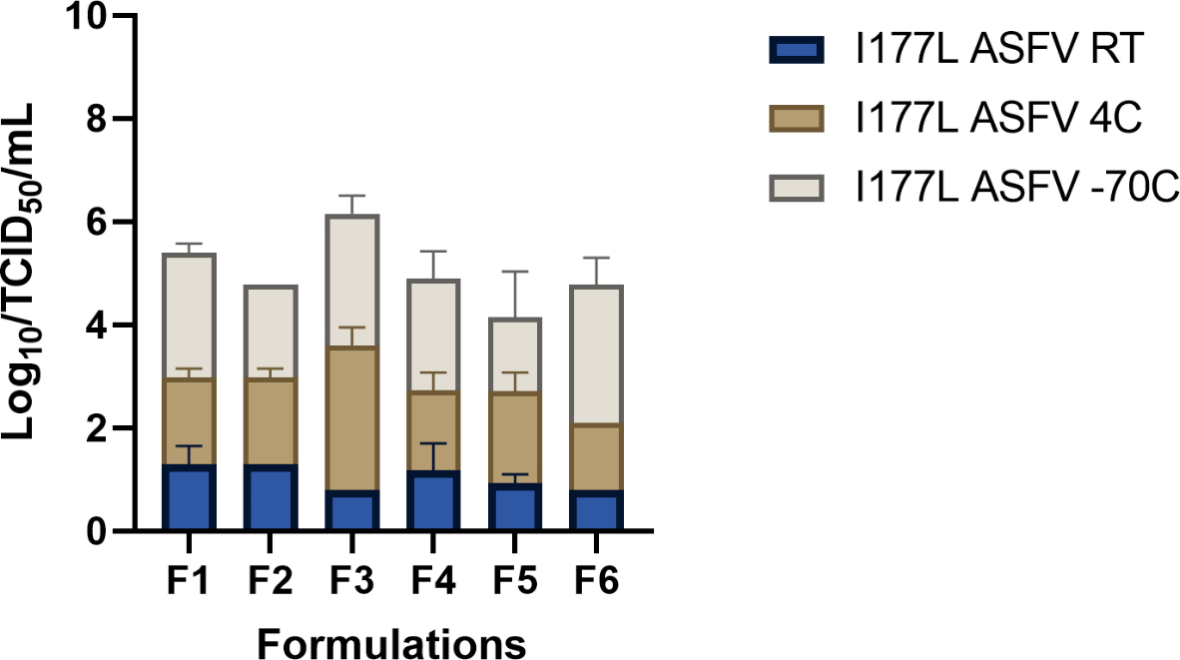
Stability of the ASFV-G-ΔI177L vaccine using lyophilization formulations using a 1:1 ratio of stablizers: vaccine indicated in Table 2, under the indicated storage conditions after three weeks. Error bars were calculated as standard error between three replica experiments.

The results of the first stabilizer screening stored in three different temperatures (Room Temperature (RT), 4 °C, and − 70 °C) after a period of three weeks (Fig. 2). All formulations were lyophilized in the same batch and with a virus-to-stabilizer ratio of 1:1.

It is important to point out that the consistency of the dried product cake formed by lyophilization collapsed for formulation F1 and F4 at RT, 4 °C, and − 70 C. On the other hand, formulations F2, F3, F5, and F6, collapsed only at 4 °C. Thus, indicating that the ratio of virus-stabilizer was not ideal and further ratio/consistency had to be tested.

Next, we tested a ratio of 1:5 using the formulations in Table 2, and we observed a better-dried consistency on the dry product was observed across all samples right after the freeze-drying process.

**Fig. 3:**
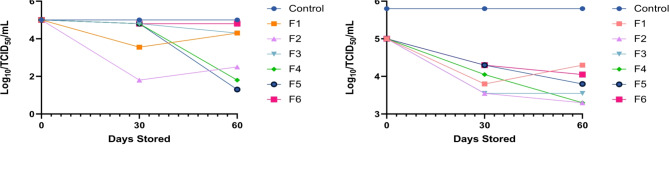
Evaluation of different lyophilization buffers listed in Table 2 using a 1:5 ratio for stability of ASFV-G-ΔI177L for short term storage at either 60 days at both 4 °C (**A**) and Room Temperature (**B**).

The viability of the vaccine candidate ASFV-G-ΔI177L was stored at two temperatures 4 C and RT, for up to sixty days (Fig. 3).

### Extended storage of lyophilized samples of ASFV-G-ΔI177L

**Fig. 4:**
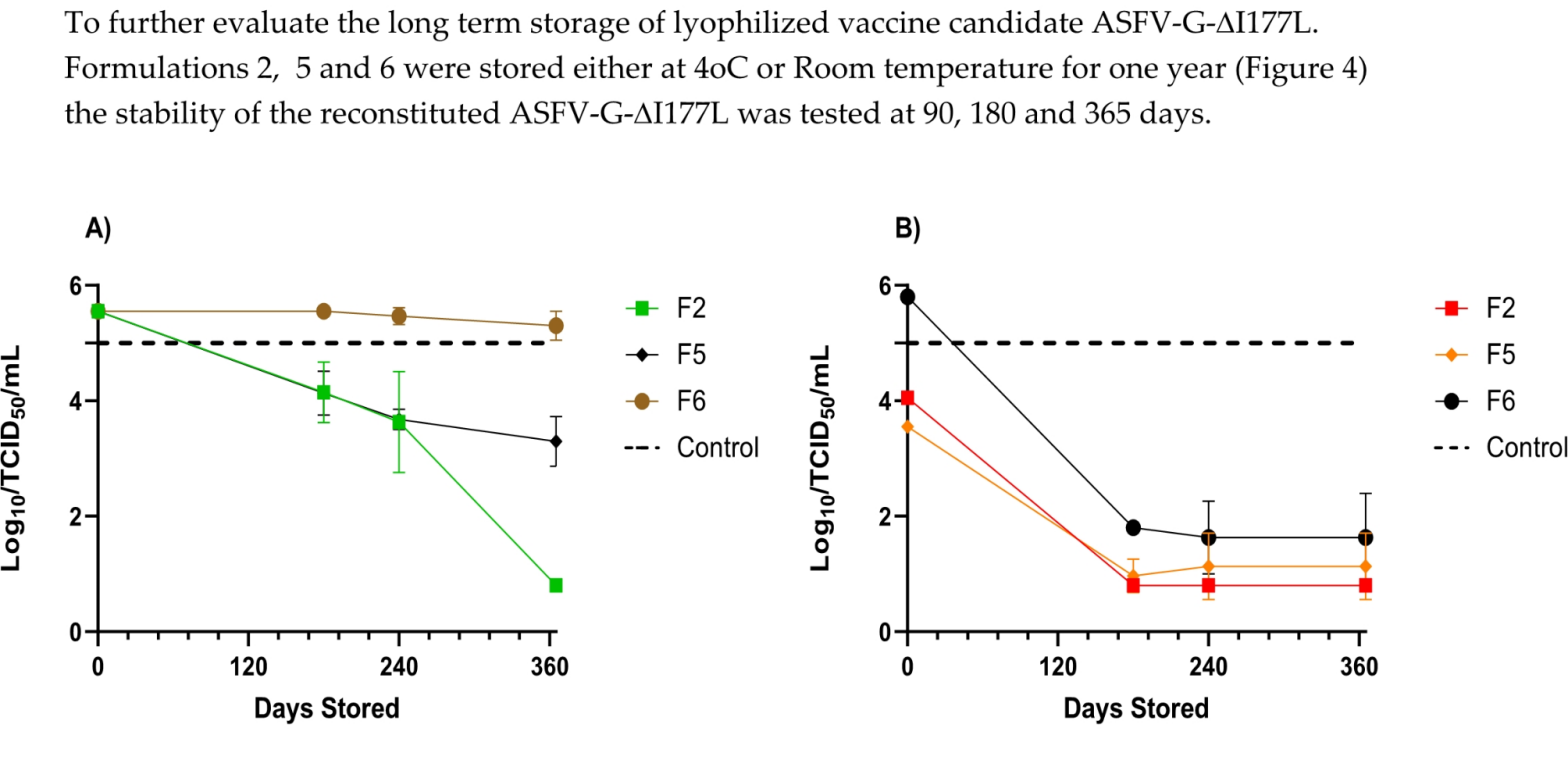
Long term stability of lyophilized vaccine under different lyophilization buffers described in Table 2 at both 4 °C (**A**) and Room temperature (**B**) storage.

To further evaluate the long term storage of lyophilized vaccine candidate ASFV-G-ΔI177L. Formulations 2, 5 and 6 were stored either at 4oC or Room temperature for one year (Fig. 4) the stability of the reconstituted ASFV-G-ΔI177L was tested at 90, 180 and 365 days.

It was observed that Formulation 6 remained stable at 4 °C for up to one year while all the other formulations had decreased stability that continued to decline over time.

Next generation sequencing was performed on ASFV-G-ΔI177L using formulation 6 buffer stored at 4 °C for 365 days. A total of 766,442 reads were aligned to the ASFV-G-ΔI177L sequence. There were no observed differences in the sequence of the lyophilized and reconstituted ASFV-G-ΔI177L genome using FR682468.2 as a reference and the same sequence was determined as in the original study^5^, indicating that lyophilized ASFV-G-ΔI177L remains stable for up to one year at 4 °C.

### Evaluation of survival rate of lyophilized for ASFV-G-∆I177L after reconstitution

**Fig. 5:**
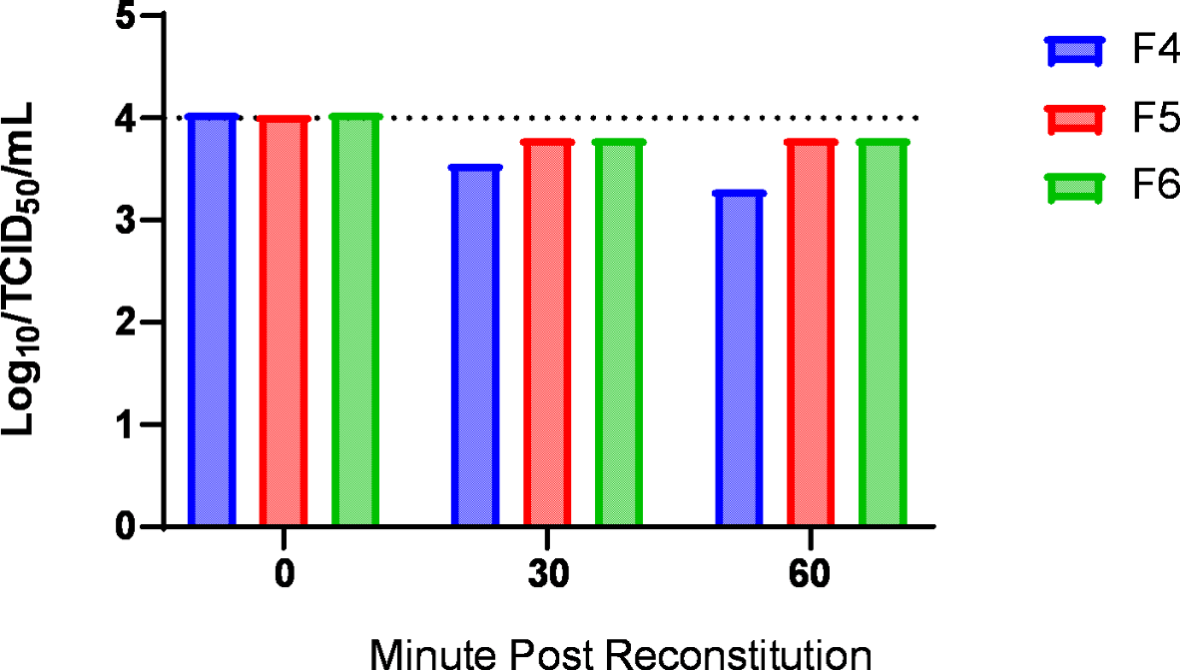
Viability was tested after reconstitution using the indicated formulas at the indicated time points.

Next, we tested the survival rate of reconstituted ASFV-G-ΔI177L for a period of one hour (60 min). The purpose of the experiment was to test the viability of ASFV-G-ΔI177L once it was reconstituted with 0.5 ml of PBS. The results suggest that ASFV-G-ΔI177L is stable for a period of 60 min with minimal titer lost (Fig. 5).

## Materials and methods

### Viruses and cells

The ASFV-G-ΔI177L vaccine stock used in this study was obtained by growth in primary swine macrophages that were isolated as previously described^10^ in T-75 Primaria flacks (Gibson, cat#156800) until complete cytopathic effect was observed. The flasks were then frozen and thawed once, and clarified by centrifugation at 3200 rmp for 10 min at 4 C. The clarified vaccine stock was then transferred to 2 ml cryotubes and stored at -70 °C.

### Freeze Drying of ASFV-G-ΔI177L for long term storage

The ASFV-G-ΔI177L vaccine was lyophilized using a FreeZone Triad Benchtop Freeze Dryers (LABCONCO, Cat# 794001010) and Rotary Vane Vacuum Pumps VACUUBRAND HYBRID (LABCONCO, Cat#7584002). Stocks of ASFV-G-ΔI177L were diluted with the indicated stabilization formulation ratio with 500ul of the stabilization buffer added to 500ul of Vaccine stock in a serum tubing vials. The freeze drier was precooled to -50°C for all lyophilization conditions tested. After lyophilization was complete and samples were maintained at 4°C until the lyophilized vials were sealed under vacuum.

### Reconstitution and evaluation of lyophilized ASFV-G-ΔI177L vaccine

After the indicated storage time lyophilized ASFV-G-ΔI177L was reconstituted with 500ul of 1X DPBS (GIBCO, Waltham, MA, USA) and vortexed briefly. Viability of lyophilized ASFV-G-ΔI177L was determine by titration on primary swine macrophages isolated fresh from donor swine blood isolated as previously described^10^. Complete cytopathic effect (CPE) was observed as the formation of rossettes as observed in ASFV infection in primary swine macrophages. Titrations were performed using the Reed and Muench method periodically^11^.

### Next generation sequencing of reconstituted lyophilized ASFV-G-ΔI177L vaccine

The reconstituted lyophilized vaccine was sequenced as previously described using an Illumina Nextseq500 sequencing platform^12^. In brief virus DNA was extracted from infected macrophage cultures with using the nuclear extraction kit (Active Motif cat# 40010) using the cytoplasmic fraction for sequencing of ASFV DNA, using Nextera XT kit (Illumnia, San Diego, CA, USA) following the manufacturer’s protocol. Sequence analysis was performed using CLC Genomics Workbench software version 24.0.2 available at https://digitalinsights.qiagen.com/products-overview/discovery-insights-portfolio/analysis-and-visualization/qiagen-clc-genomics-workbench/ (CLCBio, Waltham, MA, USA).

## Conclusion

This study is the first report evaluating lyophilization conditions for ASFV live-attenuated vaccines using ASFV-G-ΔI177L as a model. We successfully determined lyophilization conditions showing that using the stability buffer containing 10% skim milk diluted in DMEM at a ratio of 1:5 provided stability at 4 °C for one year. Interestingly, at room temperature the vaccine under all of the tested conditions had a decline in virus titers. Further studies will be required to with additional conditions to try and optimize the conditions for potential room-temperature or higher storage conditions, that may be found in some countries where ASFV has caused continued disease outbreaks.

## Data Availability

All data is available from the corresponding author.

## References

[1] Montgomery, R. E. On a form of swine fever occuring in British East Africa (Kenya Colony). *J. Comp. Pathol. Thera* **34**, 159–191 (1921).

[2] Tulman, E. R., Delhon, G. A., Ku, B. K. & Rock, D. L. African swine fever virus. In *Lesser Known Large dsDNA Viruses, Current Topics in Microbiology and Immunology* Vol. 328 (ed. Etten, V.) 43–87 (Springer, 2009). 10.1007/978-3-540-68618-7_2 19216435

[3] Chapman, D. A. et al. Genomic analysis of highly virulent Georgia 2007/1 isolate of African swine fever virus. *Emerg. Infect. Dis.* **17**, 599–605. 10.3201/eid1704.101283 (2011). 21470447 10.3201/eid1704.101283 PMC3379899

[4] Ramirez-Medina, E. et al. Experimental infection of domestic pigs with an African swine fever virus field strain isolated in 2021 from the Dominican Republic. *Viruses* **14**, 10.3390/v14051090 (2022). 35632831 10.3390/v14051090 PMC9145207

[5] Borca, M. V. et al. Development of a highly effective African swine fever virus vaccine by deletion of the I177L gene results in sterile immunity against the current epidemic Eurasia strain. *J. Virol.*10.1128/JVI.02017-19 (2020). 31969432 10.1128/JVI.02017-19 PMC7081903

[6] Tran, X. H. et al. African swine fever virus vaccine candidate ASFV-G-Δ177L efficiently protects European and native pig breeds against circulating Vietnamese field strain. *Transbound Emerg Dis*10.1111/tbed.14329 (2021). 34582622 10.1111/tbed.14329

[7] Borca, M. V. et al. ASFV-G-ΔI177L as an effective oral nasal vaccine against the Eurasia strain of Africa swine fever. *Viruses* **13**, 765. 10.3390/v13050765 (2021). 33925435 10.3390/v13050765 PMC8146859

[8] Tran, X. H. et al. Evaluation of the safety profile of the ASFV vaccine candidate ASFV-G-ΔI177L. *Viruses* **14**, 896 (2022). 35632638 10.3390/v14050896 PMC9147362

[9] Gladue, D. P. & Borca, M. V. Recombinant ASF live attenuated virus strains as experimental vaccine candidates. *Viruses* **14**, 878 (2022). 35632620 10.3390/v14050878 PMC9146452

[10] Borca, M. V., Berggren, K. A., Ramirez-Medina, E., Vuono, E. A. & Gladue, D. P. CRISPR/Cas gene editing of a large DNA virus: African swine fever virus. *Bio-protocol*10.21769/BioProtoc.2978 (2018). 34395778 10.21769/BioProtoc.2978 PMC8328649

[11] Reed, L. J. & Muench, H. A simple method of estimating fifty percent endpoints. *Am. J. Hygiene* **27**, 493–497 (1938).

[12] Borca, M. V., Holinka, L. G., Berggren, K. A. & Gladue, D. P. CRISPR-Cas9, a tool to efficiently increase the development of recombinant African swine fever viruses. *Sci. Rep.* **8**, 3154. 10.1038/s41598-018-21575-8 (2018). 29453406 10.1038/s41598-018-21575-8 PMC5816594

